# Ultraviolet A radiation and COVID‐19 deaths in the USA with replication studies in England and Italy^*^

**DOI:** 10.1111/bjd.20093

**Published:** 2021-08-01

**Authors:** M. Cherrie, T. Clemens, C. Colandrea, Z. Feng, D.J. Webb, R.B. Weller, C. Dibben

**Affiliations:** School of Geosciences University of Edinburgh EdinburghUK; School of Geosciences University of Edinburgh EdinburghUK; School of Geosciences University of Edinburgh EdinburghUK; School of Geosciences University of Edinburgh EdinburghUK; Centre for Cardiovascular Science University of Edinburgh EdinburghUK; Centre for Inflammation Research University of Edinburgh Edinburgh UK; School of Geosciences University of Edinburgh EdinburghUK

## Abstract

**Background:**

Understanding factors impacting deaths from COVID‐19 is of the highest priority. Seasonal variation in environmental meteorological conditions affects the incidence of many infectious diseases and may also affect COVID‐19. Ultraviolet (UV) A (UVA) radiation induces release of cutaneous photolabile nitric oxide (NO) impacting the cardiovascular system and metabolic syndrome, both COVID‐19 risk factors. NO also inhibits the replication of SARS‐CoV2.

**Objectives:**

To investigate the relationship between ambient UVA radiation and COVID‐19 deaths.

**Methods:**

COVID‐19 deaths at the county level, across the USA, were modelled in a zero‐inflated negative‐binomial model with a random effect for states adjusting for confounding by demographic, socioeconomic and long‐term environmental variables. Only those areas where UVB was too low to induce significant cutaneous vitamin D3 synthesis were modelled. We used satellite‐derived estimates of UVA, UVB and temperature and relative humidity. Replication models were undertaken using comparable data for England and Italy.

**Results:**

The mortality rate ratio (MRR) in the USA falls by 29% [95% confidence interval (CI) 40% to 15%) per 100 kJ m^–2^ increase in mean daily UVA. We replicated this in independent studies in Italy and England and estimate a pooled decline in MRR of 32% (95% CI 48% to 12%) per 100 kJ m^–2^ across the three studies.

**Conclusions:**

Our analysis suggests that higher ambient UVA exposure is associated with lower COVID‐19‐specific mortality. Further research on the mechanism may indicate novel treatments. Optimized UVA exposure may have population health benefits.

Seasonality^1^ and variation in temperature,^2^ humidity^3^ and ultraviolet (UV) radiation^4^ are related to the incidence of several infectious diseases. In this paper we explore whether UVA might independently affect COVID‐19 outcomes. We have previously described a novel nitric oxide (NO)‐driven, vitamin D‐independent mechanism^5^ by which sunlight can lower blood pressure, and at the population level we have shown that UV is associated with lower blood pressure^6^ and a reduced incidence of myocardial infarctions.^7^ The same UV‐driven mechanism may also cause seasonal variation in the development of diabetes and metabolic syndrome.^8^

UV may have a direct effect on the viability of SARS‐CoV2 virus in airborne droplets and on fomites,^9^ thus reducing both infection rates and the size of inoculum in those becoming infected, with correspondingly reduced disease severity^10^, ^11^ and COVID‐19 growth rates.^12^ Direct viricidal suppression of SARS‐CoV2 appears to be a UVB effect^9^ with UVA wavelengths having no effect on SARS‐CoV1.^13^ However, UVA does photo‐release NO from stores in the skin whence it is mobilized to the systemic circulation, causing vasodilatation and reduction in blood pressure,^5^ offering cardiovascular and metabolic benefits from UV exposure.^5^, ^8^ As cardiometabolic disease and possibly hypertension^14^ increase the risk of death from COVID‐19, any UV‐driven improvements in these risk factors would be expected to reduce mortality.^15^ NO may also have a specific effect on COVID‐19. It inhibits replication of SARS‐CoV^16^ and SARS‐CoV2.^17^ In the case of SARS‐CoV this occurs by S‐nitrosation of the spike protein, preventing the post‐translational palmitoylation required for fusion with its cognate angiotensin‐converting enzyme 2 receptor (ACE2R).^18^ The spike protein of SARS‐CoV is highly homologous to that of SARS‐CoV2,^19^, ^20^ suggesting that NO may similarly limit binding to ACE2R by SARS‐CoV2, accounting for reduced disease transmission and severity.

Given the apparent greater severity of illness and risk of death from COVID‐19 among those with cardiometabolic diseases,^21^, ^22^ the importance of season for infectious diseases, and a plausible pathway to reduced disease transmission and severity through photo‐released NO, we investigate whether ambient UVA exposure is associated with COVID‐19 deaths across the USA, independent of other UV pathways, and whether the finding is replicated in studies of England and Italy.

## Methods

### Study setting and participants

**Figure 1:**
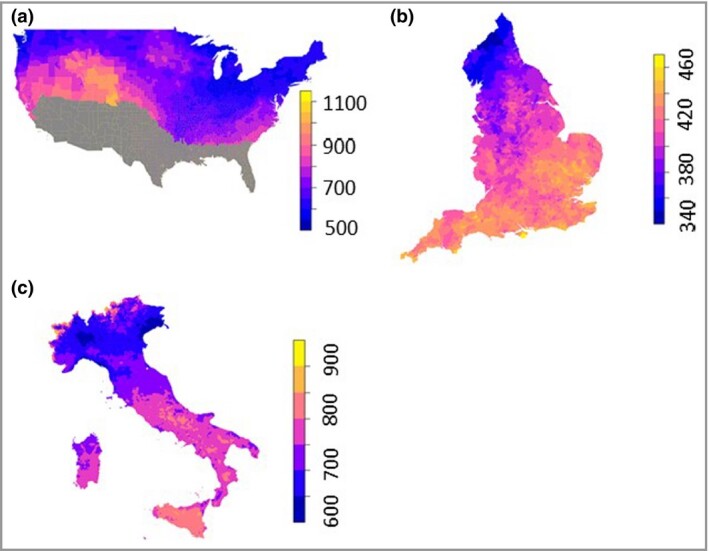
Average daily mean UVA (kJ m^–2^) January to April: (a) the USA, (b) England and (c) Italy. The UVA colour scale differs between countries. USA counties shown in grey were excluded from the study because they had monthly mean UV_vitD_ of over 165 kJ m^–2^.

We used an ecological regression approach to model COVID‐19 deaths in small areas (counties) across the contiguous USA during the early part of the COVID‐19 pandemic (January to April 2020). Our main analysis was for USA counties (*n* = 2474) with replication studies for COVID‐19 deaths across English ‘middle layer super output areas’ (MSOAs) (*n* = 6724) and excess deaths across Italian municipalities (*n* = 6775). To reduce potential confounding through a UVB vitamin D pathway [a monthly mean UV radiation on the 252–330 nm spectrum (action spectrum of UV radiation for vitamin D production, UV_vitD_, of under 165 kJ m^–2^)^23^], we included only ‘small areas’ that were experiencing levels of UV too low to induce significant cutaneous vitamin D3 synthesis at any time during the study period (‘UV vitamin D winter’). This meant that 2474 counties (out of 3088) in the USA were included within the analysis shown in Figure 1.

### COVID‐19 deaths

USA COVID‐19 deaths between 22 January and 30 April 2020 came from data compiled by the Center for Systems Science and Engineering at Johns Hopkins University.^24^ A COVID‐19 death was defined as a case if the practitioner suspected that COVID‐19 played a role, even if it was not directly attributable to the death. English COVID‐19 deaths between 1 March and 17 April 2020 were drawn from data compiled by the UK Office for National Statistics (ONS).^25^ Deaths were included if COVID‐19 was mentioned on the death certificate, with a delay of usually 5 days between occurrence and registration. In Italy there is no COVID‐19‐classified mortality data available for municipalities. Instead, we estimated this from excess deaths drawn from ISTAT (Italian Institute of Statistics)^26^ available for 7270 of 7904 municipalities for 1 March to 30 April for 2015–2019 compared with 2020.

### Ambient ultraviolet radiation

We derived mean daily UVA for the small areas in each study for these date ranges: USA (1 January – 30 April); England (1 January – 17 April) and Italy (1 January – 30 April). We start our time series of UVA before the period in which we were recording deaths because of the lag between infection and death. The UVA dataset was produced by JAXA (Japan Aerospace Exploration Agency) using the MODerate resolution Imaging Spectroradiometer (MODIS) instrument on board NASA’s Aqua and Terra satellites.^27^ Atmospheric absorption and scattering due to ozone and water (vapour and cloud) were accounted for by using a simplified planetary atmosphere (clear atmosphere positioned above a cloud layer). Downward irradiance values (i.e. combined direct and diffuse radiation on a horizontal plane) for UVA (315–400 nm) were converted to daily values by using the diurnal cycle of solar zenith angle with instantaneous atmospheric conditions. UVA data were aggregated for USA counties, English MSOAs and Italian municipalities and expressed as mean daily kJ m^–2^.

A long‐term UV_vitD_ dataset (30‐year monthly average) developed by the National Center for Atmospheric Research (NCAR) was used.^28^ UV_vitD_ data were aggregated and expressed in mean monthly kJ m^–2^. We used the highest quintile as the cutoff for year‐round vitamin D synthesis, which corresponds to a monthly mean of over 165 kJ m^–2^.^29^

### Covariates

#### Demographic and socioeconomic

A number of demographic, socioeconomic, long‐term environmental exposure and infection susceptibility variables were used in our models. This was to appropriately adjust for spatial associations, with both UVA and COVID‐19 mortality, which might otherwise lead to a spurious relationship between UVA and COVID‐19 mortality. We identified these from the existing literature and measured them at the small‐area level. Our zero‐inflated negative‐binomial (ZINB) model provides for different ways that covariates could be included. We assessed for each covariate whether it might (i) be a direct confounder for the UVA–COVID‐19 relationship, in which case they were added to the negative‐binomial (NB) part of the model and (ii) impact the exposure of individuals to the virus, in which case they were added to the zero‐inflated (ZI) part of the model. Covariates might therefore be in both parts of the model.

Older age and ethnicity were associated with higher risk of COVID‐19 death, possibly due to higher prevalence of comorbidities, including hypertension, heart disease and respiratory diseases.^30^ We used country‐specific datasets to measure risk factors associated with age and ethnicity.

Poorer citizens are at higher risk of infection due to essential working and death due to pre‐existing health conditions.^31^ We used country‐specific datasets of socioeconomic deprivation. For the USA we took the first principal component score from a principal component analysis of: percentage in poverty, median house value, median house income, percentage owner‐occupied and percentage of population with less than a high‐school education. To capture socioeconomic deprivation in England we used percentage of residents under 21 years of age who did not enter higher education and an income‐deprivation score.^32^ For Italy, we used the Italian Deprivation Index.^33^

#### Long‐term environment

Higher PM_2·5_ (fine particles with diameter ≤ 2·5 μm) exposure is linked with a range of respiratory and cardiovascular diseases and has been shown to increase COVID‐19 mortality rate in other analyses.^34^ Long‐term PM_2·5_ (2000–2016) data at a 0·01° × 0·01° resolution were modelled using satellite and monitored PM_2·5_ station data.^35^ We used these data for both the USA and Italy. In England, long‐term 2014–2018 PM_2·5_ at a 1 km × 1 km resolution was modelled using monitored PM_2·5_ station data.^36^ Variation in temperature is associated with COVID‐19 mortality.^37^ Long‐term mean monthly winter temperature (December–February) at a 4 km × 4 km resolution for 2000–2016 was modelled using satellite data^38^ for the USA. Long‐term mean monthly winter temperature (December–February) at a 1 km × 1 km resolution for 1981–2010 was modelled using interpolation of Meteorological Office weather stations for England.^39^ Long‐term median land surface temperature (December–February), daytime monthly median value at a 1 km × 1 km resolution for 2000–2017, was modelled using satellite data for Italy.^40^

#### Viral exposure

The population ‘at‐risk’ needs to be adjusted for exposure to the virus in case factors increasing or decreasing risk of exposure are associated with spatial variance in UVA levels. In densely populated, urban or peri‐urban areas, with high use of public transport, COVID‐19 transmission is faster and the prevalence of cases higher. Probable exposure is therefore estimated through population density, urban/rural status and state percentage of positive COVID‐19 tests in the USA. We used population density from the 2018 mid‐year population estimates of the UK ONS, percentage of residents using different forms of transport (bus, train, tube) from the 2011 census, and upper tier local authority (UTLA) number of days since a local authority had 10 confirmed cases in England. We used ISTAT population density from 2019, the municipality area, and total cases in province in Italy up to 30 April.^26^

### Statistical analysis

We used a ZINB regression to model counts of death in small geographical areas because the counts of deaths were likely to be spatially variable with the mean counts of deaths across small areas likely to be much smaller than the variance of the counts between the small areas and an excess of zeros (areas with no COVID‐19 infection).

We included a random effect in the model. This was a random intercept for a higher and administratively important geographical unit in each country. In the USA this was the state, in England the local authority and in Italy the province in order to: (i) capture the systematic way the risk of death from COVID‐19 might be related to this higher geography (e.g. due to differences in political administration, health services, funding, public health effects or levels of infection) and (ii) incorporate spatial clustering in the estimation of standard errors. The linearity of the relationship between UVA and COVID‐19 deaths was tested using fractional polynomial regression, which tests whether fractional powers (nonlinear) improve the fit of the model. The linear model was found to be the best fit for each country. Each of the country models were specified independently by separate team members. All models were fitted using the glmmTMB package for R,^41^ which fits random‐effect generalized linear models described in Bolker *et al*.^42^

We carried out a meta‐analysis to estimate the pooled effect across the three studies using a random‐effects model. We used a restricted maximum‐likelihood estimator with no adjustments.

#### Software

All analyses were undertaken in R 3.6.1 (https://www.r‐project.org/). We predicted the number of deaths per million population at suitable levels of UVA by calculating the marginal means in the ‘emmeans’ package in R.^43^ We used a random‐effects model as part of the ‘metafor’ package in R^44^ to calculate a cross‐county pooled estimate of the mortality rate ratio (MRR).

#### Data and code

Data and code are presented in the following online repository: https://github.com/markocherrie/COVID19_UVA

## Results

Daily mean UVA (January–April 2020) varied between 340 and 1000 kJ m^–2^ across the three countries, with lower average levels experienced across England (340–460 kJ m^–2^) during the period compared with Italy (600–900 kJ m^–2^) and USA (500–1000 kJ m^–2^) (Figure 1a–c). For the USA the average county UVA level between January and 30 April was 696 kJ m^–2^ (SD 83). For England the average MSOA UVA level between January and 17 April was 412 kJ m^–2^ (SD 18). For Italy the average UVA level between January and 30 April was 717 kJ m^–2^ (SD 52).

**Figure 2:**
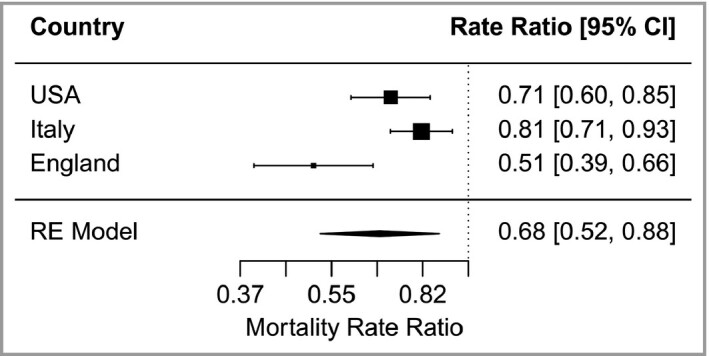
Mortality rate ratios per 100 kJ m^–2^ increase in mean daily UVA [pooled estimate from random effects (RE) model].

We model the rate of COVID‐19 deaths in small areas in a multilevel ZINB model for the USA, England and Italy (Table 1). The model estimate is the MRR per 100 (kJ m^–2^) increase in UVA or the ratio of the mortality rate in a small area with a similar area exposed to 100 (kJ m^–2^) more UVA. Adjusting for other confounders measured at the small‐area level (county, MSOA and municipality) and adjusting for infection rates at a higher level of geography (state, UTLA and province) (Table 2) our estimates show reductions in MRR of 0·71 in the USA per 100 kJ m^–2^ increase in UVA (Figure 2). We found a similar size of effect in our two replication studies: an MRR in Italy of 0·81 and in England 0·51. For the random‐effects meta‐analysis across the three countries we find a pooled MRR estimate of 0·68 per 100 increase in UVA (kJ m^–2^) (Figure 2).

**Table 1:** Zero‐inflated negative‐binomial models for the USA, England and Italy showing the change in COVID‐19 mortality rate ratio per 100 kJ m^–2^ increase in UVA

Model^a^/Country	Geographic units of analysis	Random effect	Deaths, *n*	Adjusted MRR (95% CI) per 100 kJ m^–2^ increase in UVA	ICC
USA	Counties (*n* = 2474)	State (*n* = 46)	62 219 (COVID‐19 deaths)	0·71 (0·60–0·85)	0·042
England	MSOAs (*n* = 6724)	UTLA (*n* = 150)	19 315 (COVID‐19 deaths)	0·51 (0·39–0·66)	0·231
Italy	Municipalities (*n* = 6775)	Province (*n* = 104)	46 095 (excess deaths)	0·81 (0·71–0·93)	0·068

CI, confidence interval; ICC, intraclass correlation coefficient; MRR, mortality rate ratio; MSOA, middle layer super output areas; UTLA, upper tier local authority; UVA, ultraviolet A radiation.

^a^ Adjusted for the variables shown in Table 2.

**Table 2:** Models adjusted for these variables

Model / Country	Geographic units of analysis	Negative‐binomial model, adjusted for	Zero‐inflated model, adjusted for
USA	County level	PM_2·5_ UV_vitD_ Winter temperature Winter humidity Percentage of residents: 65+ years, black, Hispanic Deprivation score Urban/rural	Percentage of residents: 65+ years, black, Hispanic Deprivation score Urban/rural
	State level	Proportion of positive COVID‐19 cases	Proportion of positive COVID‐19 cases
England	MSOA level	PM_2·5_ Long‐term winter temperature Percentage of residents: 80+ years, 65–79 years, black, Indian, Pakistani/Bangladeshi, Chinese, in care homes, in higher education Income‐deprivation score	Percentage of residents: 80+ years, 65–79 years, black, Indian, Pakistani/Bangladeshi, Chinese, in care homes, in higher education, using public transport (bus, train, tube) Income‐deprivation score Population density
	UTLA level	Number of days since a local authority had 10 confirmed cases	Number of days since a local authority had 10 confirmed cases
Italy	Municipalities level	PM_2·5_ Long‐term winter temperature Number of foreign born Percentage of residents: 65+ years, 85+ years Population density Municipality area Deprivation score	Number of foreign born Percentage of residents: 65+ years, 85+ years Population density Municipality area Deprivation score
	Province level	Total cases in province	Total cases in province

MSOAs, middle layer super output areas; PM_2·5_, fine particles with diameter ≤ 2·5 μm; UTLA, upper tier local authority.

**Figure 3:**
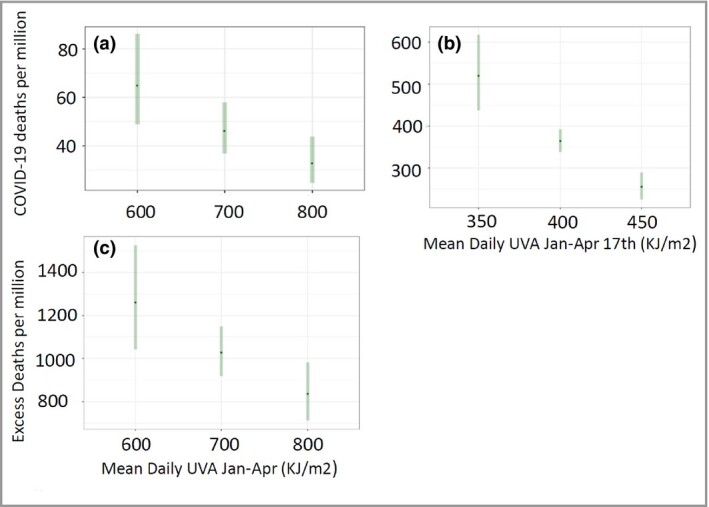
Predicted COVID‐19 rates of deaths at selected levels of UVA in (a) the USA, (b) England and (c) Italy, given the model random effect, at the mean level of all other covariates. The predicted risks reflect the pandemic situation (i.e. infection levels) in each country at the time of the study.

Using the model (including the random effects) we predict what the rate of mortality (deaths per million population) would be for different experienced levels of UVA (all other coefficients in the model held at their means) in the three countries (Figure 3a–c). This illustrates that the models predict a similar approximate fall by a third to a half in the average risk of death across the levels of UVA experienced across the three countries. This is despite the very different levels of death being experienced between the countries at this point in the epidemic. The models also suggest that the size of the risk reduction per 100 (kJ m^–2^) UVA is largest for England which has the lowest average level of UVA during the period.

## Discussion

Our analysis finds that higher ambient UVA exposure is associated with lower COVID‐19‐specific mortality. This effect appears independent of differences in socioeconomic composition, temperature, humidity and UV within the vitamin D action spectrum. Our models are consistent with a situation where UVA exposure may be an additional UV‐protective factor against COVID‐19, along with other potential UV‐related pathways through vitamin D production and direct viricidal suppression. This is an observational study and so further research is required.

Given that UVA does not appear to directly act in viricidal suppression of SARS‐CoV2,^9^, ^13^ the reduction in the observed risk might be the result of either behavioural change or a biological pathway. Warm and sunny days might increase the chance of time spent outdoors or away from indoor spaces where close contact with others would be higher. Although UVA and air temperature are associated, in our models we also control for temperature. Our findings are consistent between three countries and with varying latitudes. It seems unlikely that time spent outdoors would be sufficiently linearly associated with UVA for the same calendar period, after controlling for temperature, over such a wide range of latitude and UVA to produce the same effect on time spent outdoors (e.g. between Newcastle and Naples in March). In contrast, there is increasing evidence that UVA photo‐releases NO from skin and that NO has important potential impacts on virus replication.^16^–^18^ NO S‐nitrosates the spike protein of SARS‐CoV and also the SARS‐CoV and SARS‐CoV2 protease. This double action blocks the myristolation of the spike protein necessary for it to bind to ACE2R, and also inhibits viral protease activity, with consequent reduced viral replication and cytopathic effects.^16^–^18^ Maintenance of the health vasculature is dependent on constitutive endothelial NO synthase‐derived NO. Endothelial damage and excess coagulation may underlie widespread organ involvement^45^ in COVID‐19, which would be mitigated by photochemical NO production. Although further research is required, we suggest that this NO biological pathway is a plausible explanation for our model results. UVA also correlates inversely with population blood pressure and cardiovascular disease^6^, ^7^ and associates with reduced all‐cause mortality, largely through a reduction in cardiovascular deaths.^46^

Tolerance to UVA, as shown by the reduced fall in COVID‐19 deaths we demonstrated for a given incremental rise in UVA at higher irradiances in the USA and Italy, compared with England, could be explained by increasing melanin in the skin blocking UV penetration. Adaptive pigmentation occurs even at relatively low levels of UVA, with approximately half of the annual variation in forehead pigmentation for white‐skinned individuals living in Copenhagen (55·68° N) occurring between January and April.^47^

Deaths from COVID‐19 are disproportionately high in people of black African or Asian ancestry in Europe and the USA.^48^, ^49^ Social factors probably account for much of this, but we have shown that the vitamin D and temperature‐independent fall in blood pressure with increased UV is attenuated in black compared with white Americans.^6^ White skin colour is an evolutionary adaptation that occurred around 5000 years ago in Neolithic agriculturalists migrating to low‐light northern Europe.^50^ Darker skin colour would thus be anticipated to reduce any biological benefits from UV, whether blood pressure reduction or infection prevention, particularly at higher latitudes. The remarkably low COVID‐19 mortality in equatorial Africa is consistent with this.^51^

There are of course weaknesses in our design. We have adjusted our models for all the clinically significant factors that we believe might be spatially or temporally associated with both UVA and COVID‐19 mortality risk but if any unmeasured factors exist, they might plausibly explain the relationships identified. UVA and covariates are measured at the small‐area level not in individuals. We therefore assume the unmeasured individual‐level model is replicated in the ecological model and this may not be the case. However, it is unclear what relationship between UVA and different geographical areas could exist that would modify its relationship with COVID‐19. Ambient UVA is likely to be a poor measure of individual‐level UVA level exposure (e.g. exposure could be mainly derived from indoor environments). However, in an ecological model the risk associated with personal exposure is averaged across all people in a small area and, therefore, the ‘mismeasurement’ of personal UVA exposure is predominantly Berkson error. This type of error will not bias an ecological model, unless the error is associated, at the small‐area level, with ambient UVA level. For this to systematically occur in our models seems unlikely. Satellite‐derived UVA measures may be overestimated under high aerosol loadings (e.g. when the PM_2·5_ count is high) and this may mean that our model, because we adjust for PM_2·5_, underestimates the association between actual UVA exposure and COVID‐19 deaths. There could be misclassification of deaths and rates of infection are estimated within the model and with indirect measures. However, any resulting measurement errors seem unlikely to be correlated with spatial variation in UVA and therefore biasing. The random effect in our models will incorporate differences between socially and politically distinct regions (states, local authorities and municipalities) that might induce a spurious relationship between UVA and mortality. The replication of the findings across three countries with very different health systems, economic and political structures, pandemic situations and climates suggests a robust finding.

In conclusion, this study is observational and therefore any causal interpretation needs to be taken with caution. However, if the relationship identified proves to be causal, it suggests that optimizing sun exposure may be a possible public health intervention. Given that the effect appears independent of a vitamin D pathway, it suggests possible new COVID‐19 therapies and the importance of exploring the role of circulating NO.

## Acknowledgments

Thank you Hiwot Weller for filming the video summary.

## Author Contribution

**Mark Cherrie:** Formal analysis (equal); Methodology (equal); Writing‐original draft (equal); Writing‐review & editing (equal). **Tom Clemens:** Formal analysis (equal); Methodology (equal); Writing‐original draft (equal). **Claudio Colandrea:** Formal analysis (equal); Writing‐original draft (equal). **Zhiqiang Feng:** Formal analysis (equal); Writing‐original draft (equal). **David J Webb:** Methodology (equal); Writing‐review & editing (equal). **Richard Beresford Weller:** Conceptualization (equal); Investigation (equal); Methodology (equal); Project administration (equal); Supervision (equal); Writing‐original draft (equal); Writing‐review & editing (equal). **Chris Dibben:** Conceptualization (equal); Formal analysis (equal); Funding acquisition (equal); Investigation (equal); Methodology (equal); Project administration (equal); Supervision (equal); Writing‐original draft (equal); Writing‐review & editing (equal).

Author video.

Video content can be viewed at https://onlinelibrary.wiley.com/doi/10.1111/bjd.20093

## Supplementary Material

bjd20093-sup-0001-Journal_Club **Powerpoint S1** Journal Club Slide Set.
