# Supplementary material for: Ultraviolet A radiation and COVID‐19 deaths in the USA with replication studies in England and Italy*
Source: Br J Dermatol. 2021 Aug 1;185(2):363–70. doi: 10.1111/bjd.20093 (PMC8251104; doi:10.1111/bjd.20093)
Supplement: bjd20093-sup-0001-Journal_Club — Powerpoint S1 Journal Club Slide Set. [file bjd20093-sup-0001-journal_club.pptx]

## Slide 1
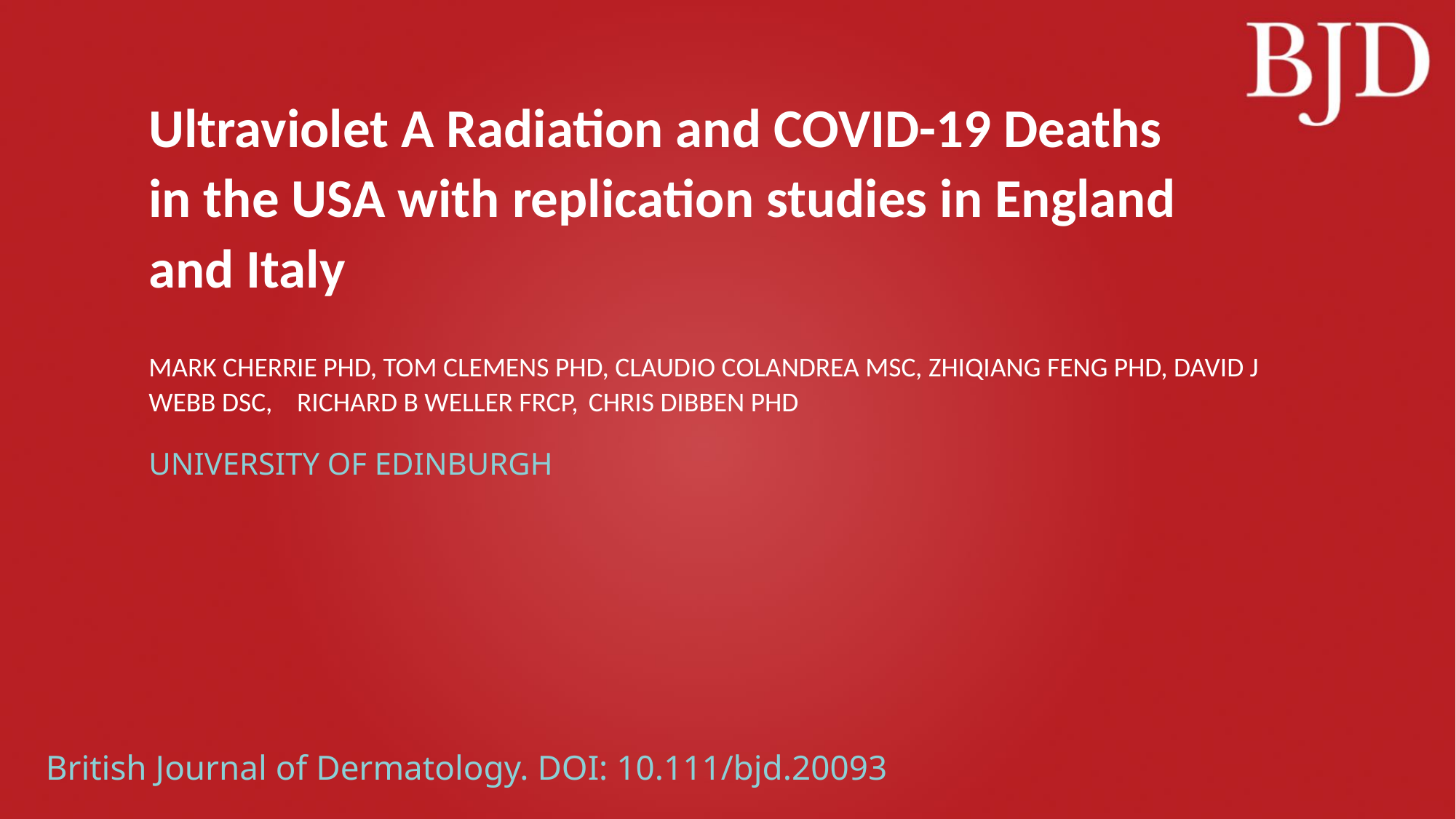

# Ultraviolet A Radiation and COVID-19 Deaths in the USA with replication studies in England and Italy
Mark Cherrie PhD, Tom Clemens PhD, Claudio Colandrea MSc, Zhiqiang Feng PhD, David J Webb DSc, Richard B Weller FRCP, Chris Dibben PhD
University of EDINBURGH
British Journal of Dermatology. DOI: 10.111/bjd.20093

## Slide 2
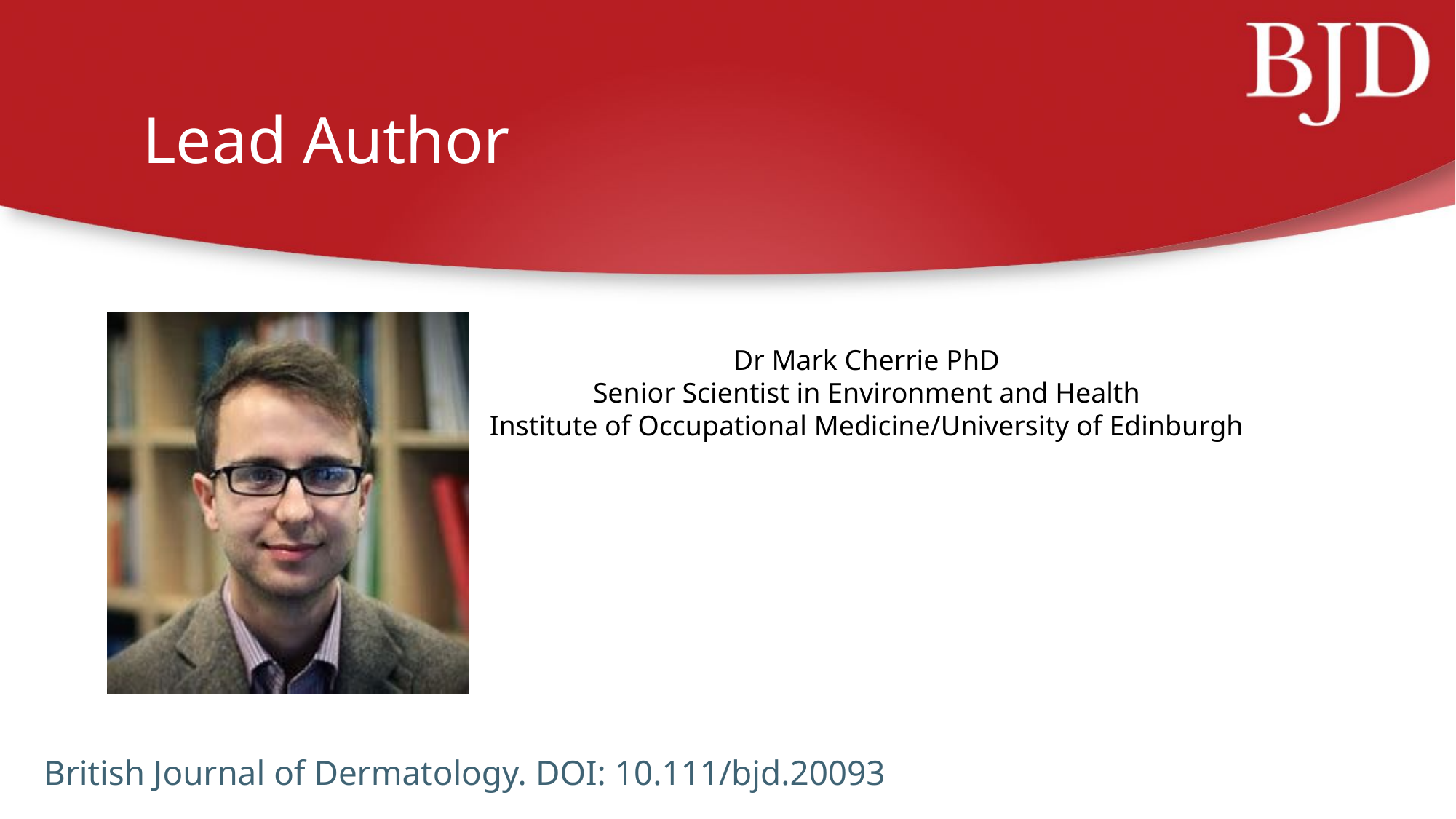

# Lead Author
Dr Mark Cherrie PhD
Senior Scientist in Environment and Health
Institute of Occupational Medicine/University of Edinburgh
British Journal of Dermatology. DOI: 10.111/bjd.20093

## Slide 3
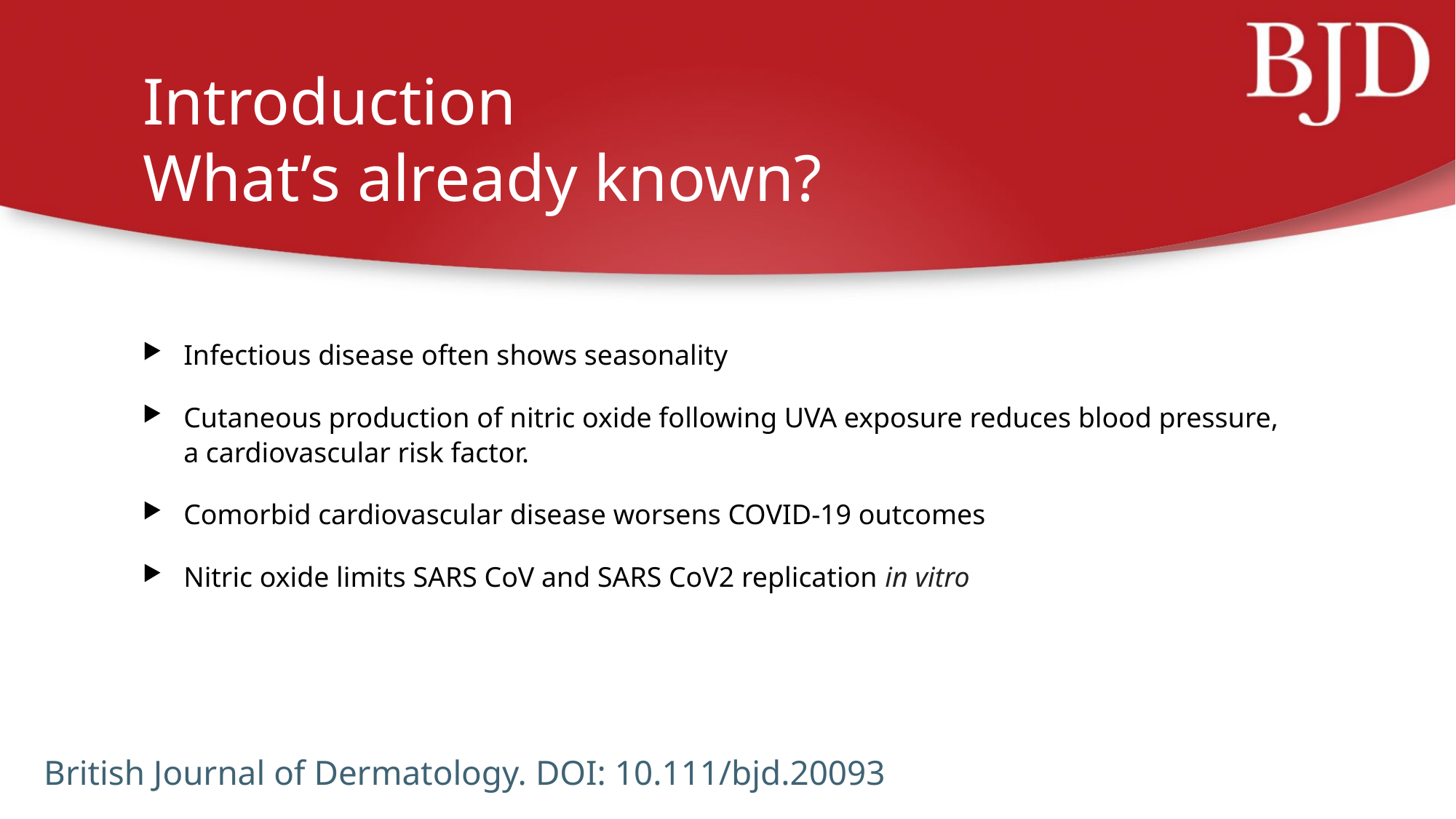

# Introduction What’s already known?
Infectious disease often shows seasonality
Cutaneous production of nitric oxide following UVA exposure reduces blood pressure, a cardiovascular risk factor.
Comorbid cardiovascular disease worsens COVID-19 outcomes
Nitric oxide limits SARS CoV and SARS CoV2 replication in vitro
British Journal of Dermatology. DOI: 10.111/bjd.20093

## Slide 4
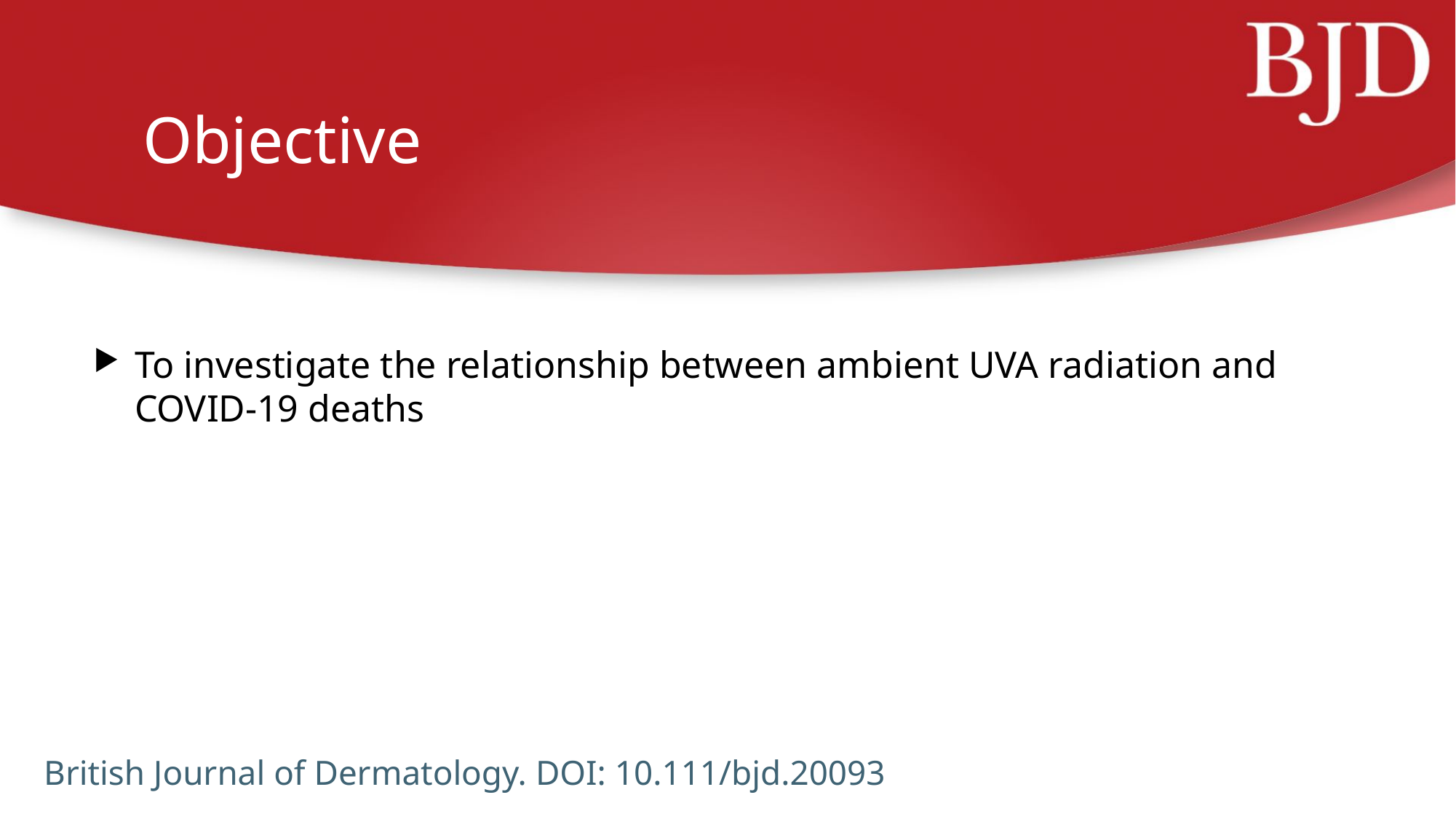

# Objective
To investigate the relationship between ambient UVA radiation and COVID-19 deaths
British Journal of Dermatology. DOI: 10.111/bjd.20093

## Slide 5
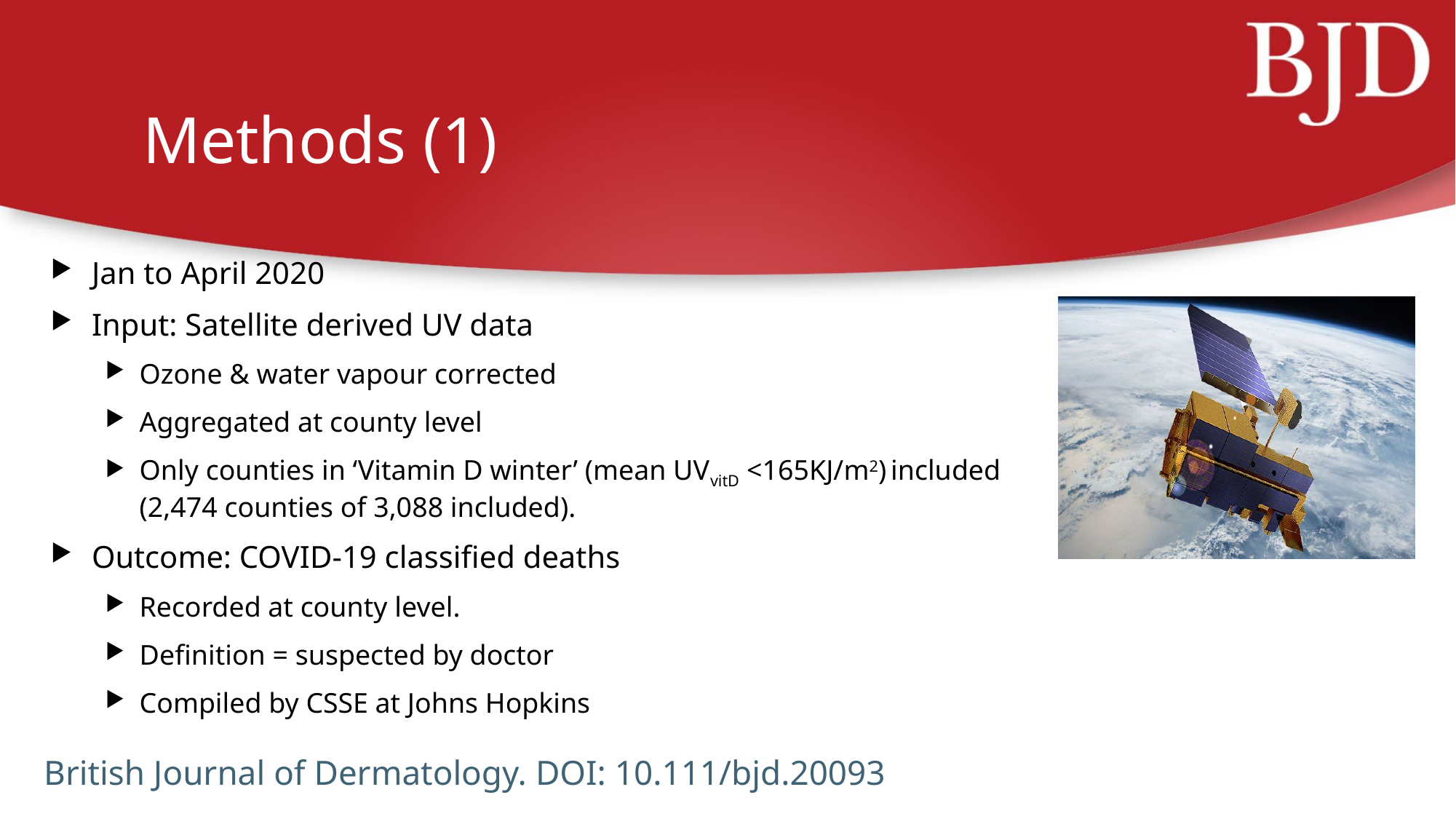

# Methods (1)
Jan to April 2020
Input: Satellite derived UV data
Ozone & water vapour corrected
Aggregated at county level
Only counties in ‘Vitamin D winter’ (mean UVvitD <165KJ/m2) included (2,474 counties of 3,088 included).
Outcome: COVID-19 classified deaths
Recorded at county level.
Definition = suspected by doctor
Compiled by CSSE at Johns Hopkins
British Journal of Dermatology. DOI: 10.111/bjd.20093

## Slide 6
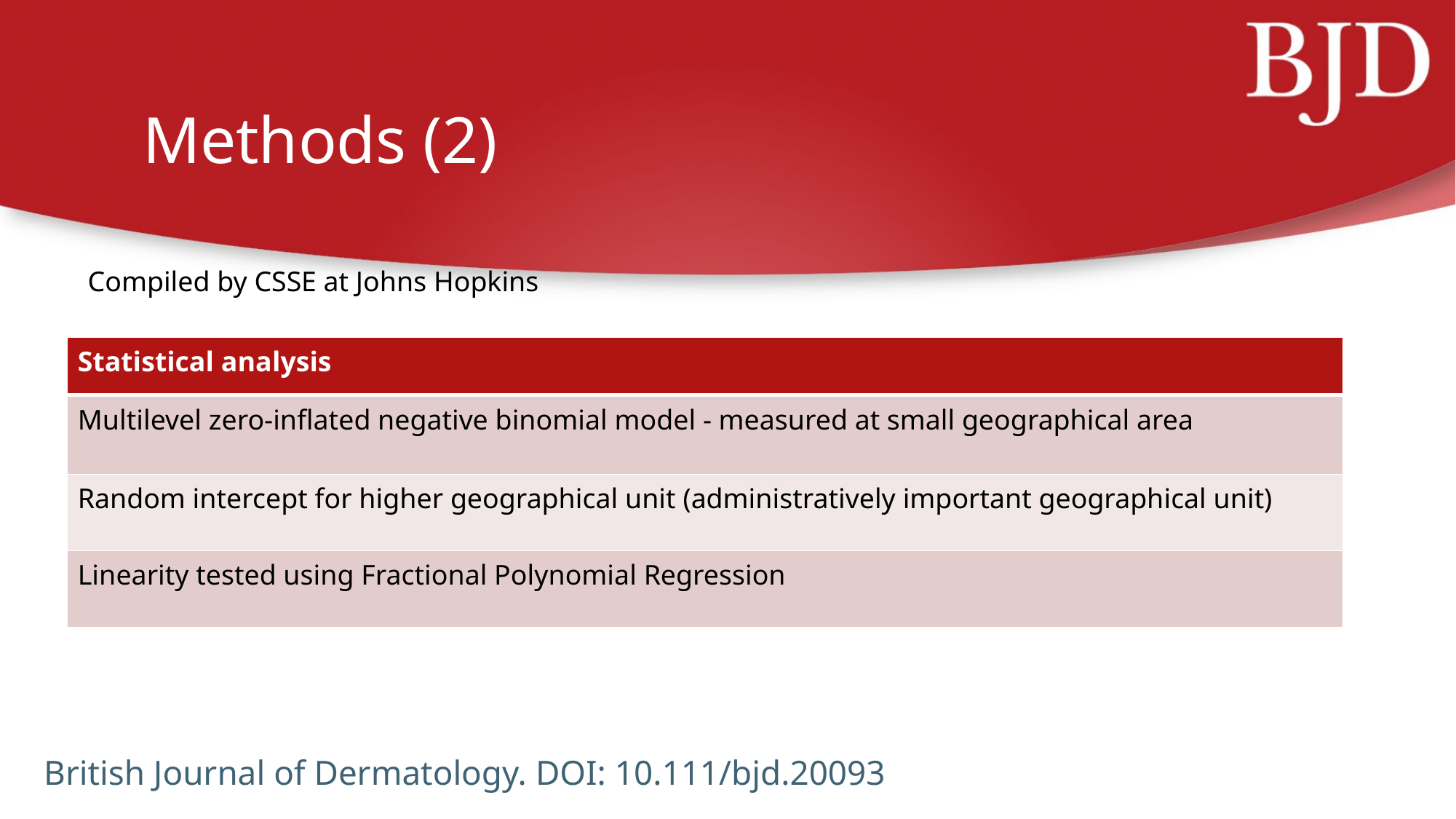

# Methods (2)
Compiled by CSSE at Johns Hopkins
Infection susceptibility
| Statistical analysis |
| --- |
| Multilevel zero-inflated negative binomial model - measured at small geographical area |
| Random intercept for higher geographical unit (administratively important geographical unit) |
| Linearity tested using Fractional Polynomial Regression |
British Journal of Dermatology. DOI: 10.111/bjd.20093

## Slide 7
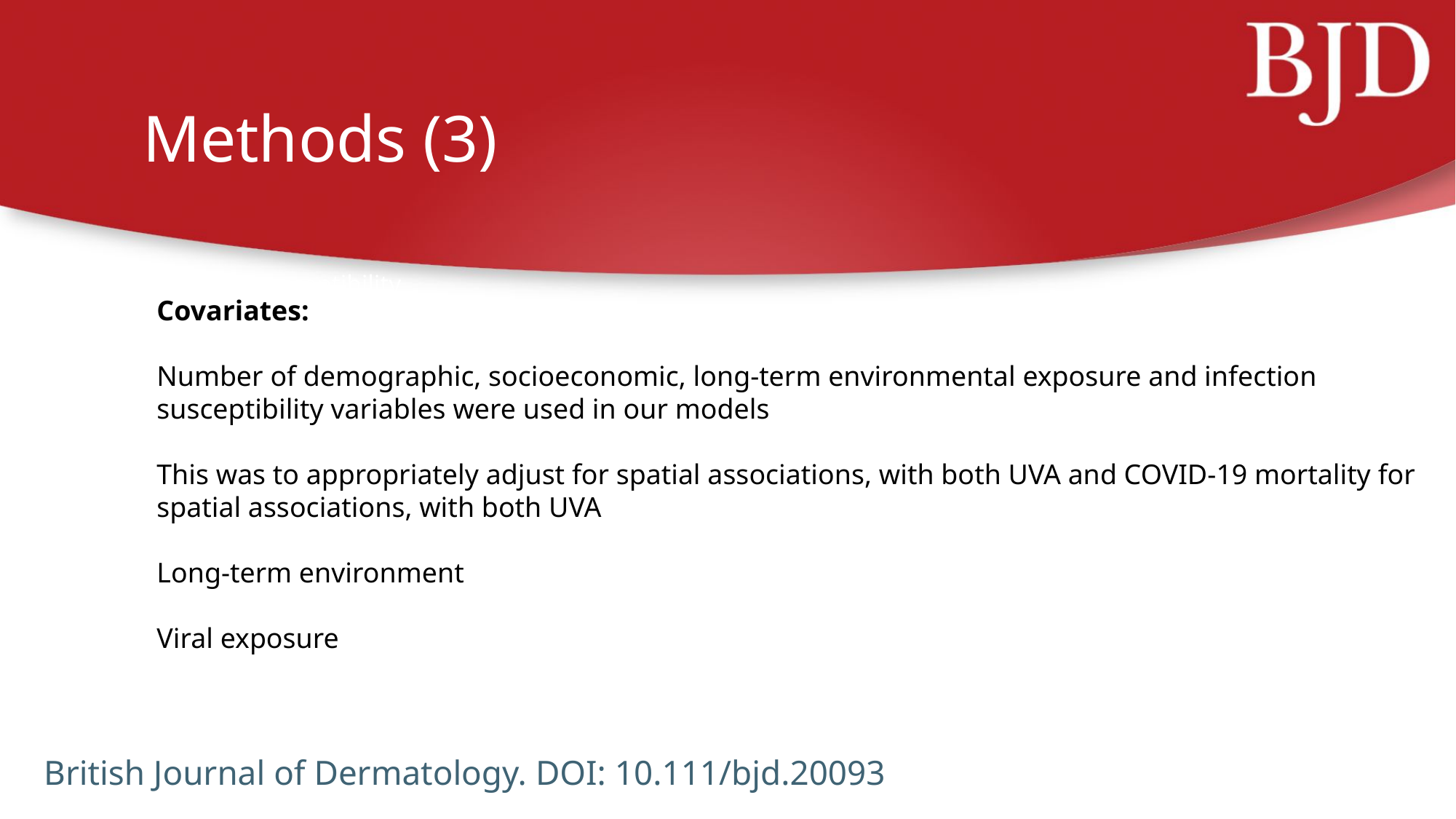

# Methods (3)
Infection susceptibility
Covariates:
Number of demographic, socioeconomic, long-term environmental exposure and infection susceptibility variables were used in our models
This was to appropriately adjust for spatial associations, with both UVA and COVID-19 mortality for spatial associations, with both UVA and COVID-19 mortality, which might otherwise lead to
Long-term environment
Viral exposure
British Journal of Dermatology. DOI: 10.111/bjd.20093

## Slide 8
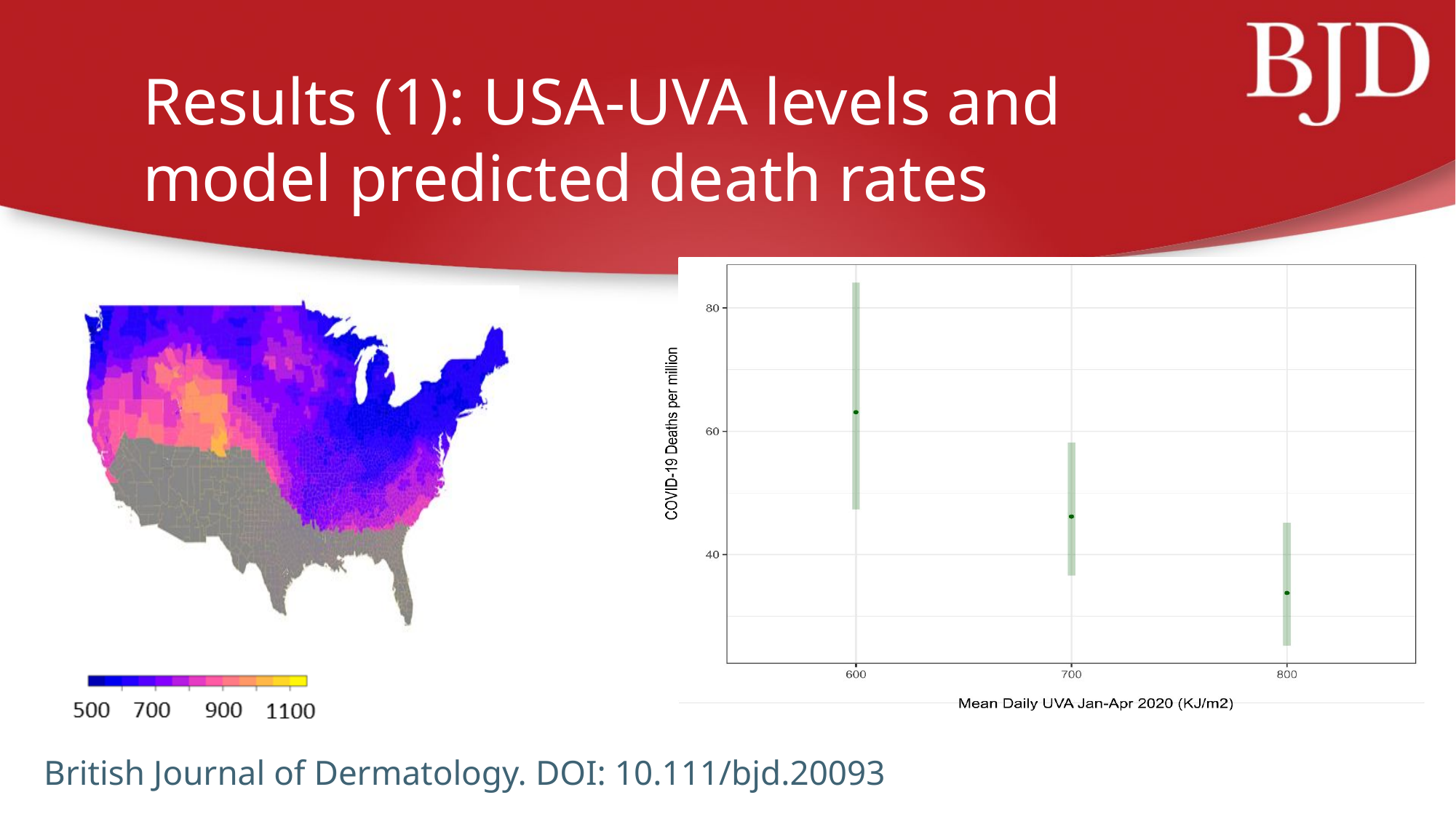

# Results (1): USA-UVA levels and model predicted death rates
British Journal of Dermatology. DOI: 10.111/bjd.20093

## Slide 9
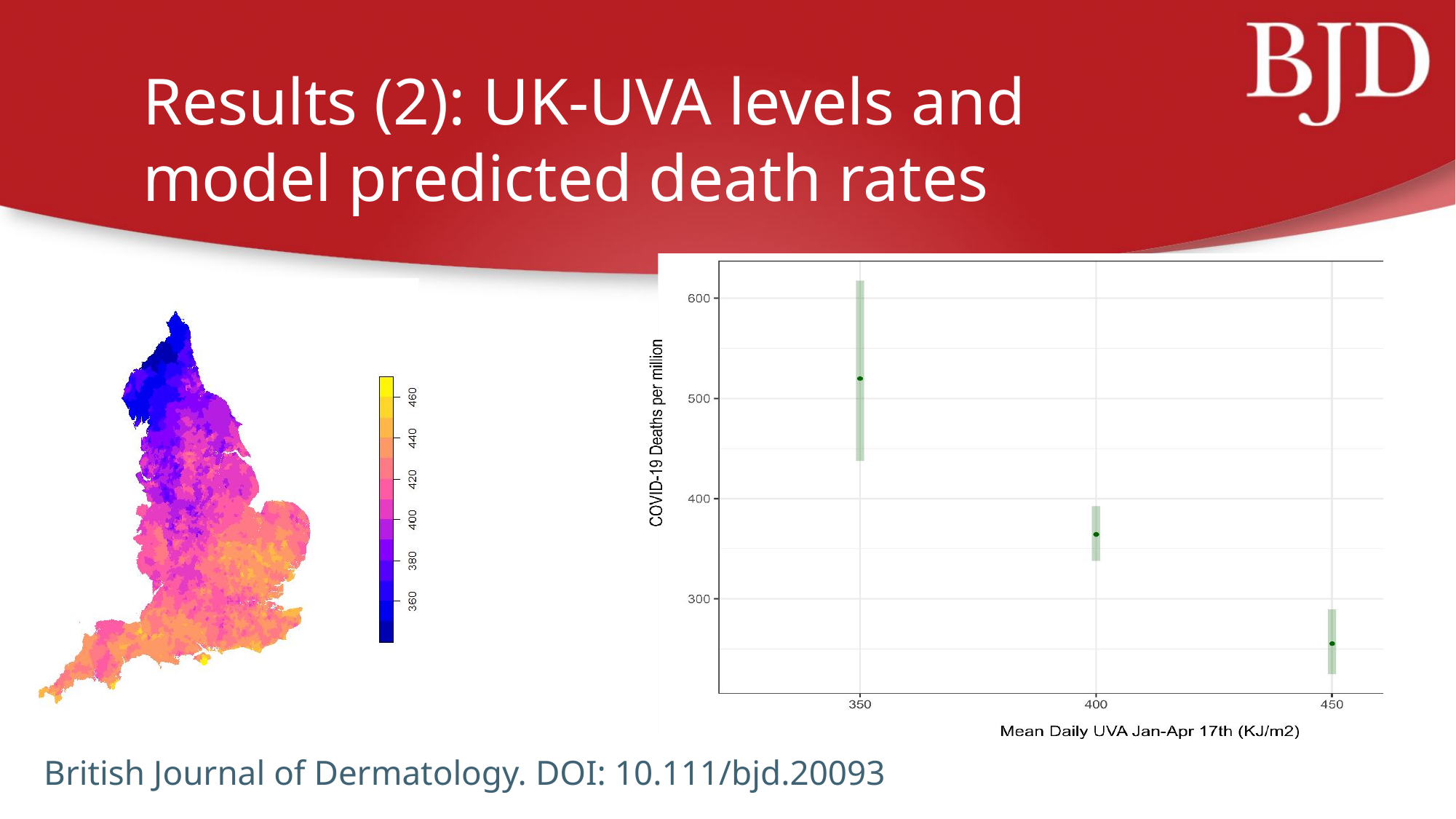

Results (2): UK-UVA levels and model predicted death rates
British Journal of Dermatology. DOI: 10.111/bjd.20093

## Slide 10
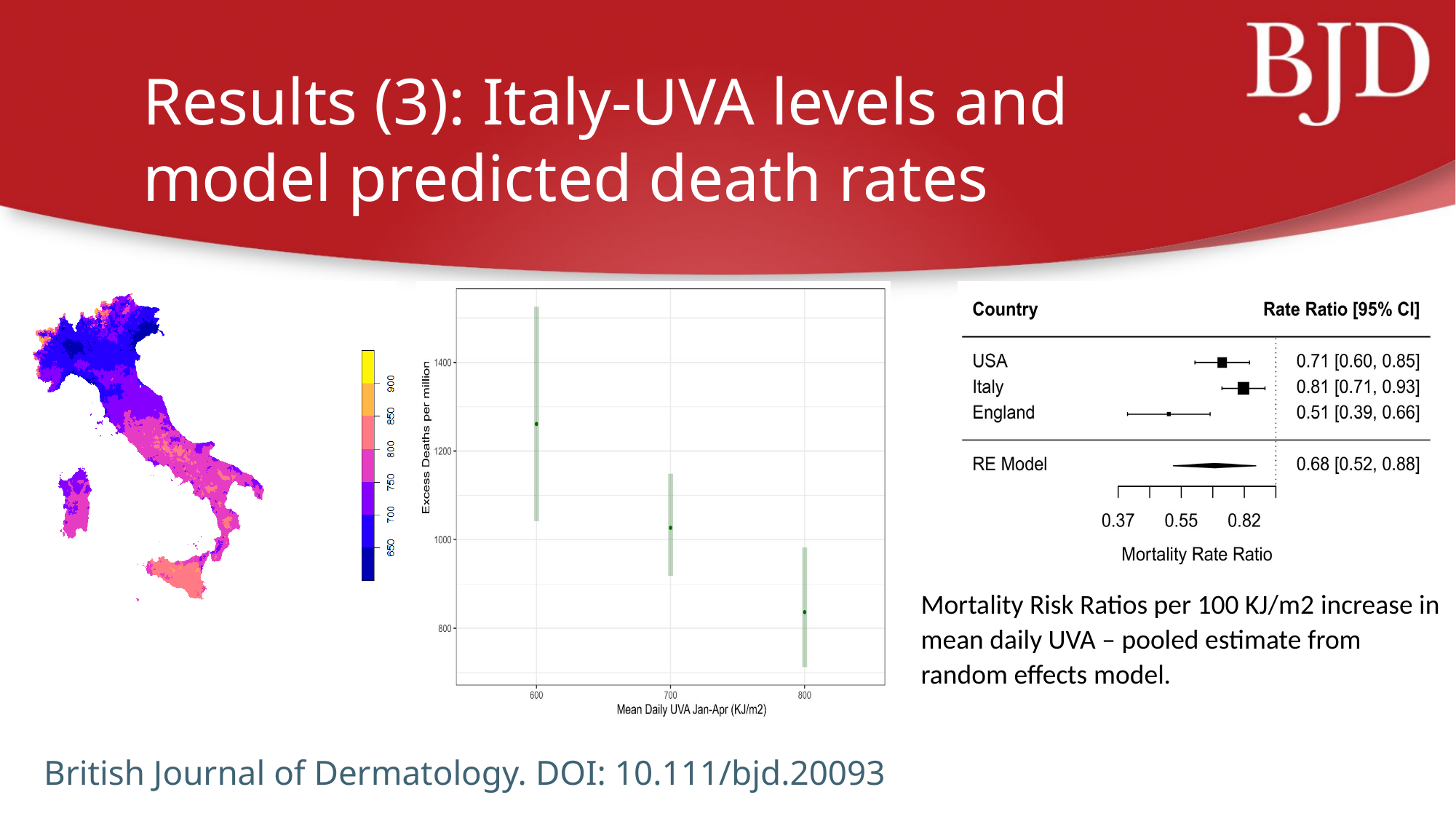

Results (3): Italy-UVA levels and model predicted death rates
Mortality Risk Ratios per 100 KJ/m2 increase in mean daily UVA – pooled estimate from random effects model.
British Journal of Dermatology. DOI: 10.111/bjd.20093

## Slide 11
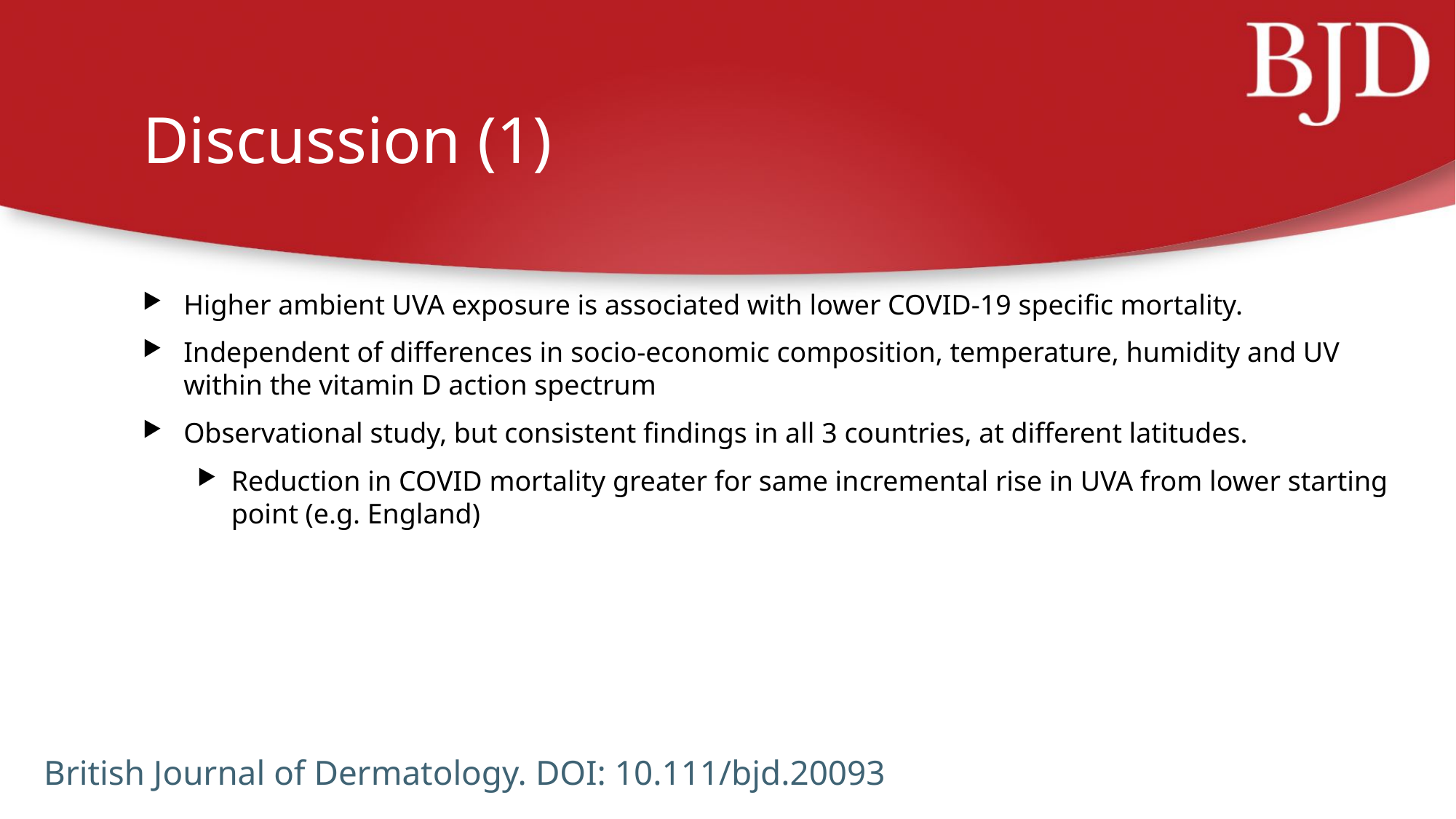

# Discussion (1)
Higher ambient UVA exposure is associated with lower COVID-19 specific mortality.
Independent of differences in socio-economic composition, temperature, humidity and UV within the vitamin D action spectrum
Observational study, but consistent findings in all 3 countries, at different latitudes.
Reduction in COVID mortality greater for same incremental rise in UVA from lower starting point (e.g. England)
British Journal of Dermatology. DOI: 10.111/bjd.20093

## Slide 12
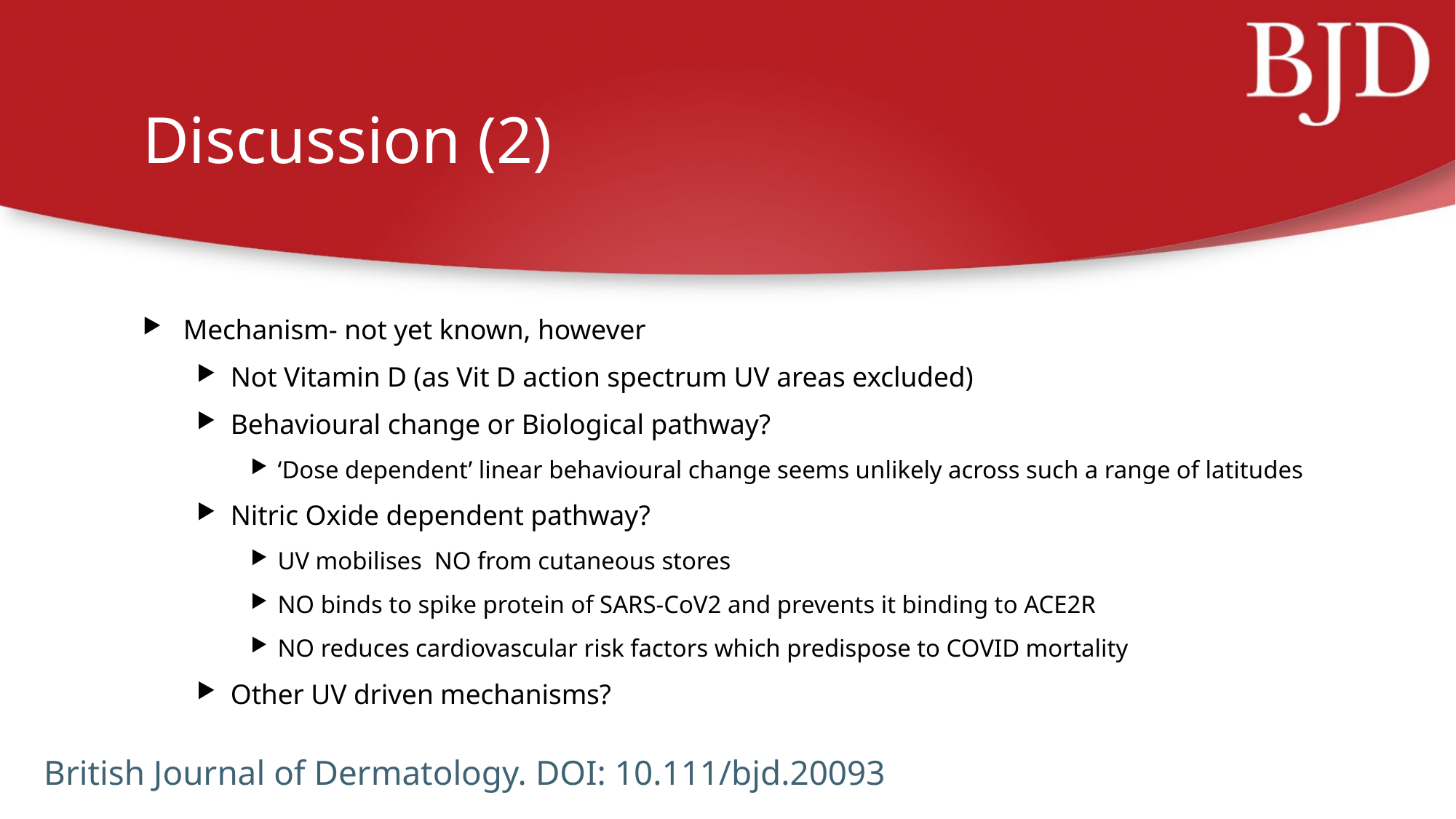

# Discussion (2)
Mechanism- not yet known, however
Not Vitamin D (as Vit D action spectrum UV areas excluded)
Behavioural change or Biological pathway?
‘Dose dependent’ linear behavioural change seems unlikely across such a range of latitudes
Nitric Oxide dependent pathway?
UV mobilises NO from cutaneous stores
NO binds to spike protein of SARS-CoV2 and prevents it binding to ACE2R
NO reduces cardiovascular risk factors which predispose to COVID mortality
Other UV driven mechanisms?
British Journal of Dermatology. DOI: 10.111/bjd.20093

## Slide 13
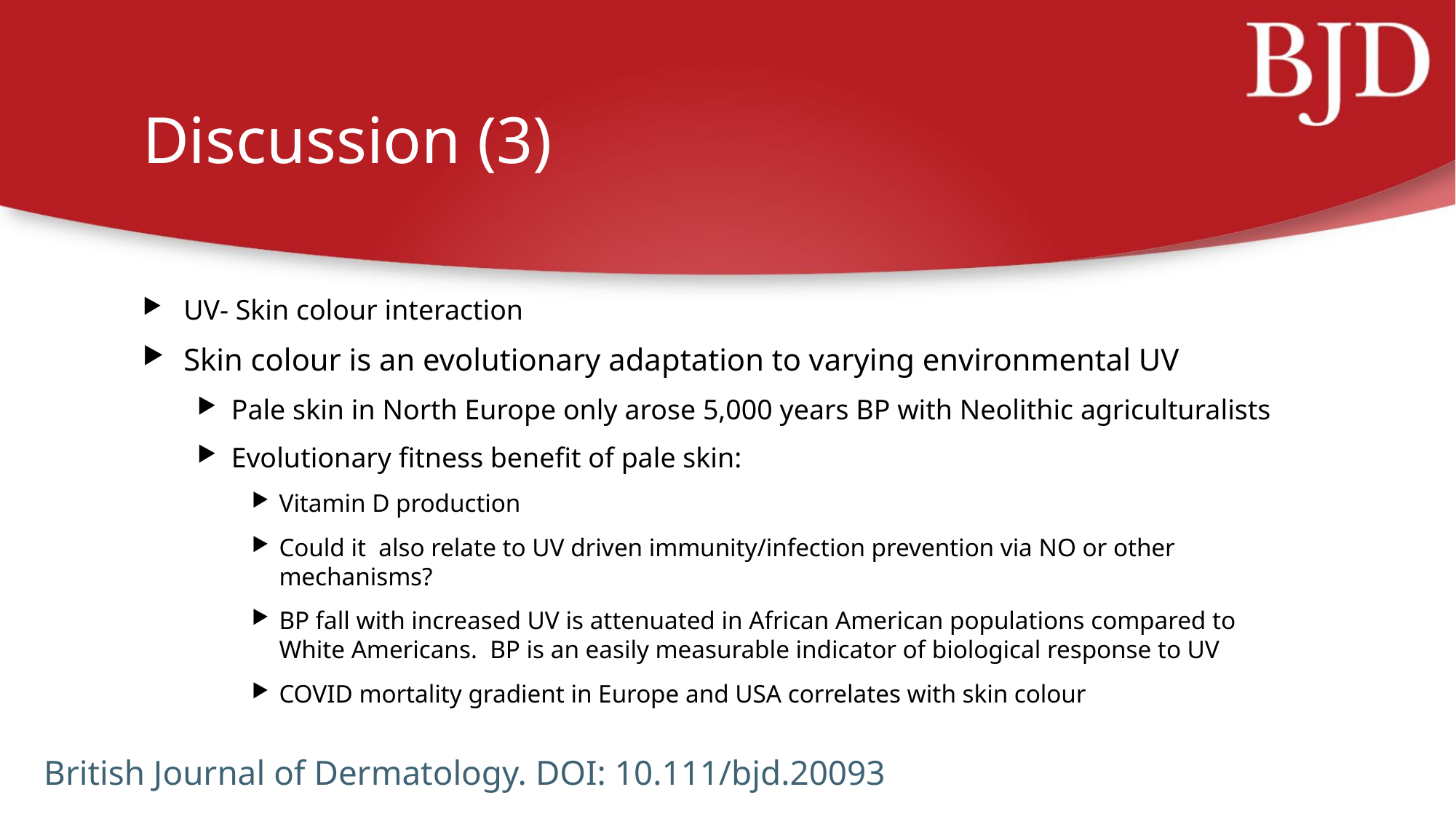

# Discussion (3)
UV- Skin colour interaction
Skin colour is an evolutionary adaptation to varying environmental UV
Pale skin in North Europe only arose 5,000 years BP with Neolithic agriculturalists
Evolutionary fitness benefit of pale skin:
Vitamin D production
Could it also relate to UV driven immunity/infection prevention via NO or other mechanisms?
BP fall with increased UV is attenuated in African American populations compared to White Americans. BP is an easily measurable indicator of biological response to UV
COVID mortality gradient in Europe and USA correlates with skin colour
British Journal of Dermatology. DOI: 10.111/bjd.20093

## Slide 14
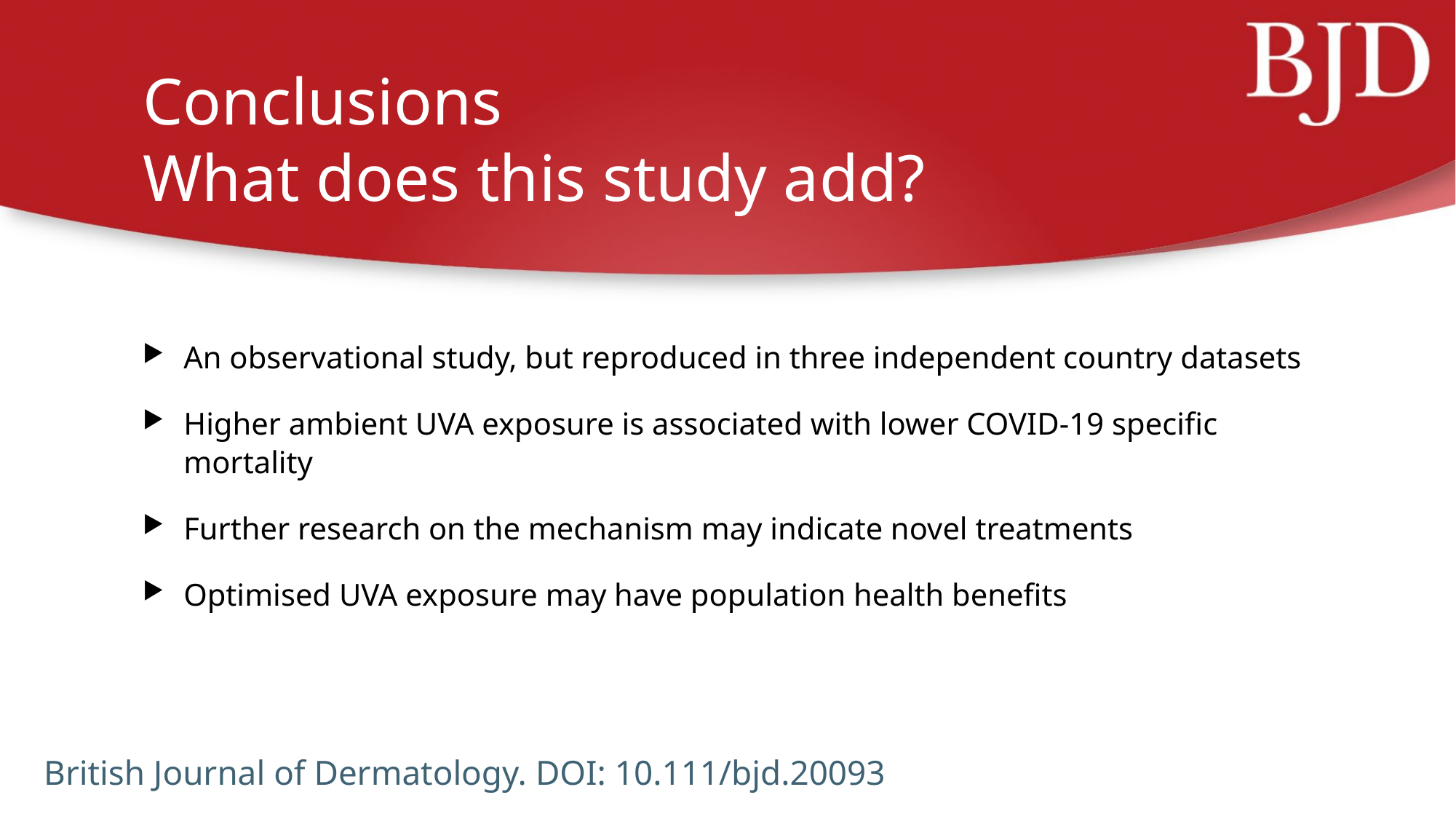

# Conclusions What does this study add?
An observational study, but reproduced in three independent country datasets
Higher ambient UVA exposure is associated with lower COVID-19 specific mortality
Further research on the mechanism may indicate novel treatments
Optimised UVA exposure may have population health benefits
British Journal of Dermatology. DOI: 10.111/bjd.20093

## Slide 15
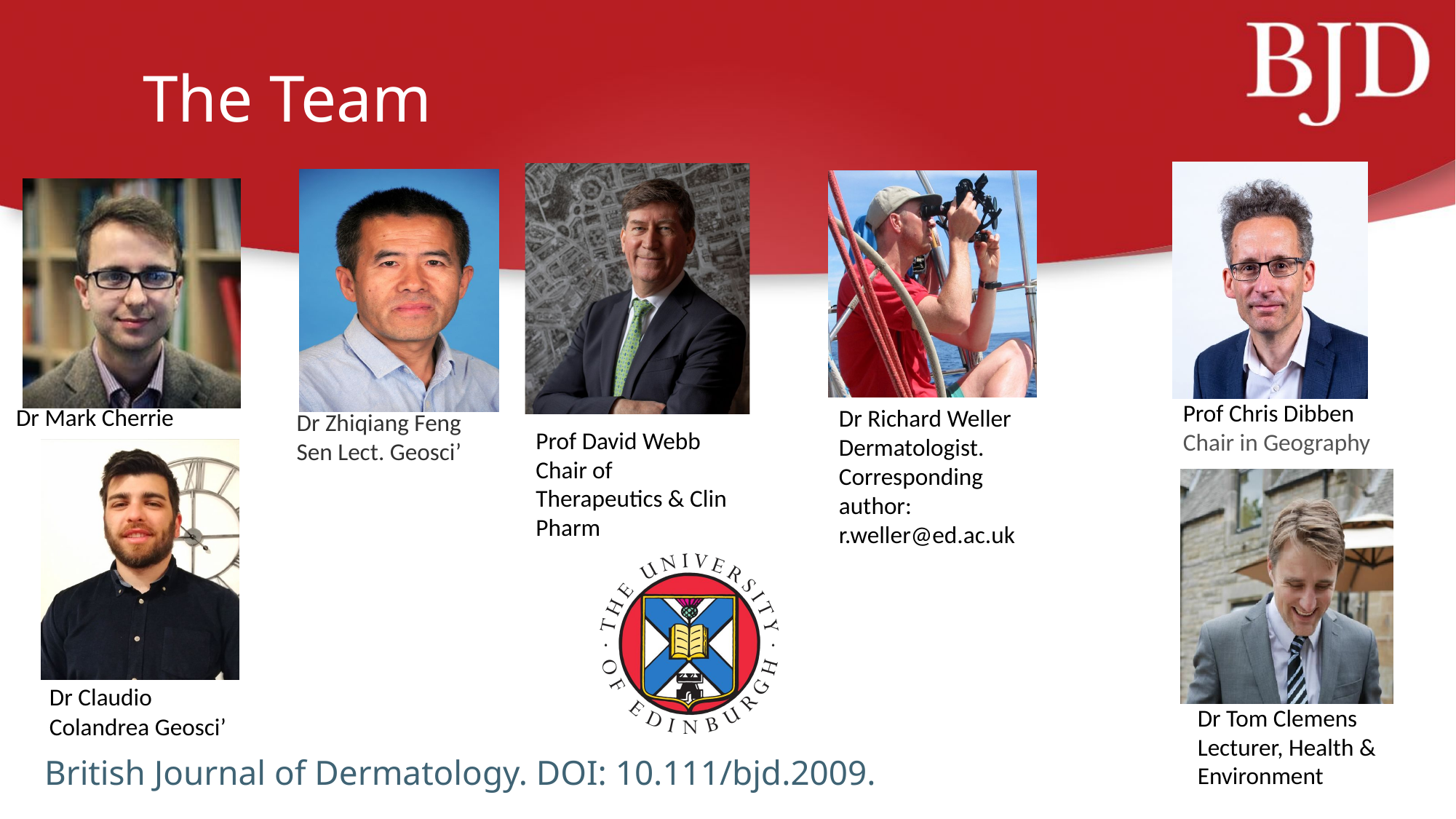

# The Team
Prof Chris Dibben
Chair in Geography
Dr Zhiqiang Feng
Sen Lect. Geosci’
Dr Richard Weller
Dermatologist. Corresponding author: r.weller@ed.ac.uk
Dr Mark Cherrie
Prof David Webb
Chair of Therapeutics & Clin Pharm
Dr Claudio
Colandrea Geosci’
Dr Tom Clemens
Lecturer, Health & Environment
British Journal of Dermatology. DOI: 10.111/bjd.2009.

## Slide 16
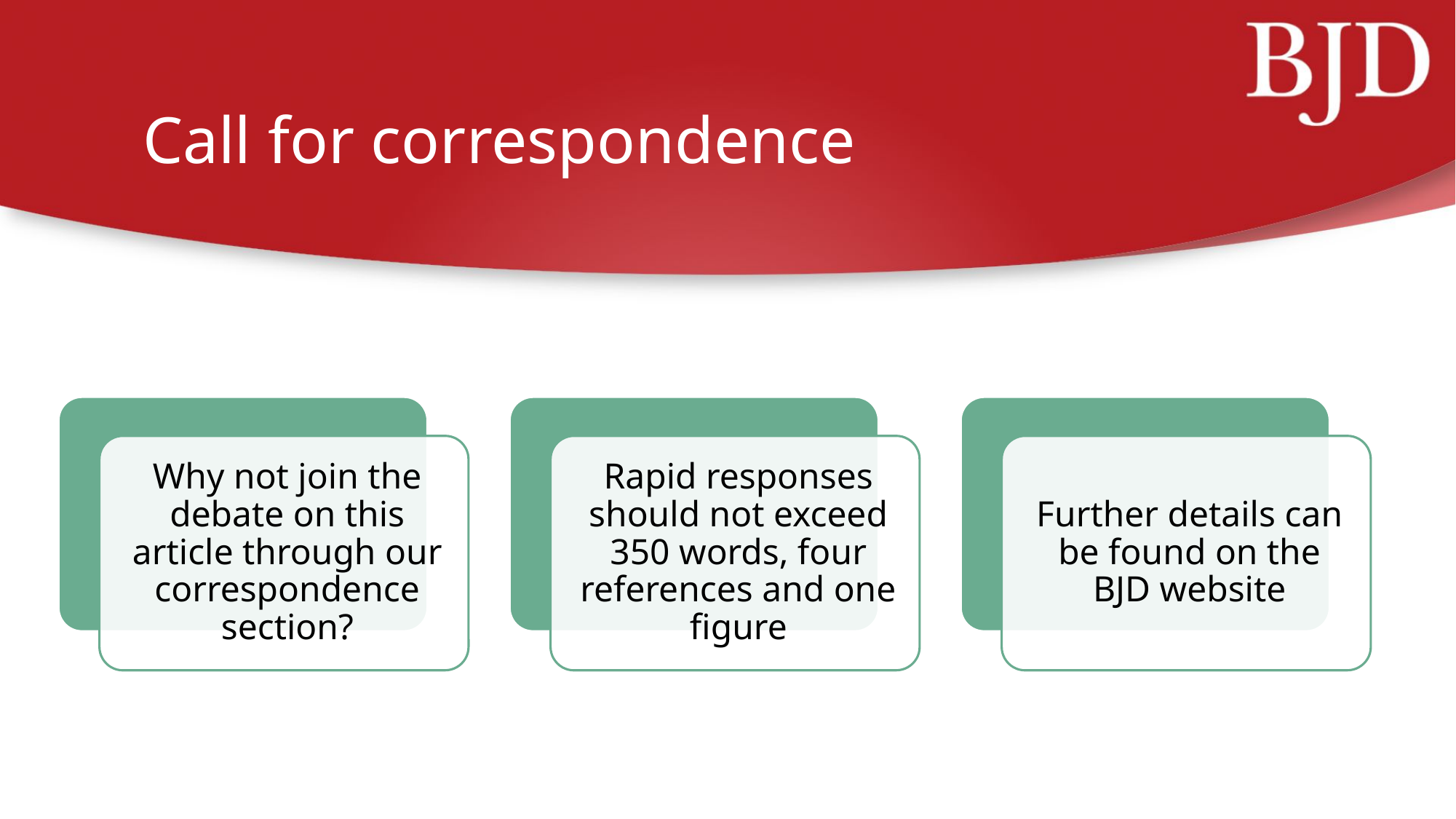

# Call for correspondence
